# What Can Mathematics Do for Drug Development?

**DOI:** 10.1007/s11538-019-00632-x

**Published:** 2019-07-22

**Authors:** Helen Moore, Richard Allen

**Affiliations:** 1 Oncology R&D, AstraZeneca, Waltham, MA 02451 USA; 2 Present Address: Applied Mathematics, Applied BioMath, Concord, MA 01742 USA; 3 Internal Medicine Research Unit, Pfizer Inc, Cambridge, MA 02139 USA

In a blog post from July 2017, the US Food and Drug Administration (FDA) Commissioner Scott Gottlieb emphasized the importance of modeling and simulation (M&S) in drug development (Gottlieb 2017). He wrote, “FDA’s Center for Drug Evaluation and Research (CDER) is currently using modeling and simulation to predict clinical outcomes, inform clinical trial designs, support evidence of effectiveness, optimize dosing, predict product safety, and evaluate potential adverse event mechanisms.” He also wrote about the benefits of M&S in individualizing therapies, in predicting dose adjustments in patients with renal impairment, and in predicting responses in patients with rare diseases, which are hard to study in clinical trials due to the small numbers of patients.

These examples reflect a growing awareness in industry of ways that M&S can support and advance our understanding of both pharmacology and biology. Historically, the biotechnology/pharmaceutical (biopharma) industry M&S focus has been on pharmacology, in the form of pharmacokinetic and pharmacodynamic modeling (Rowland and Tozer 2011). The growing field of quantitative systems pharmacology (QSP) takes a more biologically mechanistic, systems view to mathematically describe diseases and potential therapies. Application of QSP modeling has been more widely adopted within industry over the last fifteen years (Nijsen et al. 2018).

Given the industry and regulatory recognition of the value of M&S, particularly in describing complex biological systems and disease, the mathematical biology community has many opportunities to help drug development. Most work used in submissions to the FDA and other regulatory agencies is performed by modelers working in the biopharma industry. However, there are many academics whose expertise and interests are aligned with these problems; they are in a position to have meaningful impact for large numbers of patients. In this special issue of the Bulletin for Mathematical Biology, *Mathematics to Support Drug Discovery and Development*, we present a collection of papers with this common theme. Our goal in collecting these papers is to provide detailed examples of techniques used in the biopharma industry, in a journal read by many mathematical biology modelers in academia.

Modelers working in the biopharma industry, including the two of us, have an interest in building stronger ties between industry and academia. Our perspectives article (Allen and Moore 2019) discusses some particular ideas for this, including attending conferences we don’t usually attend and publishing in different journals. For example, journals commonly read by modelers in biopharma include the Journal of Pharmacokinetics and Pharmacodynamics (JPKPD) and CPT:Pharmacometrics & Systems Pharmacology (CPT:PSP). In our article, we also include additional methods that are commonly used or that we would like to be used in the biopharma industry.

Some of the papers in this issue reflect an emphasis seen in the industry on predicting drug concentrations more accurately. More accurate predictions mean we can select doses with higher confidence, which can reduce the use of animals and the risk to patients. Improvements in accuracy can come through improved mathematical and numerical methods, or through incorporating additional mechanistic detail. Wu et al. (2019) analyze a mathematical model for drug concentrations after multiple doses of a drug that is cleared by both linear and nonlinear routes simultaneously and is also produced endogenously. They determine a closed-form analytical solution for these complex dynamics and use that to examine questions of interest about longer-term behavior. Saltzman and Bendtsen (2018) develop a novel model for drug absorption by incorporating advection, diffusion, and binding of a drug in the lining of the intestines. Their analysis yields measurable criteria that can be used in drug development to determine drug transit across the mucin lining. Vendel et al. (2018) start with a model for drug transport across the blood–brain barrier and incorporate two-dimensional drug transport and binding within brain tissue to better predict drug concentrations in the brain.

Two of the papers focus on drug binding kinetics. The paper of Naganawa et al. (2017) considers positron emission tomography (PET) image data for the binding of drugs to their intended target. The authors apply maximum likelihood statistical methods to improve the quantification of PET image binding data with the goal of minimizing bias and variance and show results for both simulated and real data. White and Bridge (2018) formulate and analytically solve differential equation models for drugs with one- and two-ligand binding kinetics. Their dynamical systems analysis provides conditions that indicate when the dose–response curves will be multiphasic.

Other papers focus on modeling therapeutic drug effects with additional mechanistic details. Lemaire and Cox (2018) refine their previous model for the dynamics of osteoblasts and osteoclasts in bone remodeling by incorporating additional mechanistic pathways. They use this refined model to explore the effects of specific therapies on different pathways and assess the results by comparing to data. Karolak and Rejniak (2018) construct a two-dimensional model for drug transport within tumor tissue by advection and diffusion and for binding to target receptors. They build a tool that can customize treatment based on tissue characteristics measured in digitized images of patient tumor tissue.

All of the topics described above are approached using ordinary differential equations or partial differential equations. Terms describing “local” behavior are used to incorporate mechanistic information about a biological system into the mathematical model. The equations are typically analyzed for general behavior or solved numerically to determine system-level behavior. The other two papers in this issue both look at networks, and problems in drug discovery that can benefit from different types of network analyses.

The paper of Gnacadja (2017) presents two open mathematical problems in pharmacology. The first open problem is to systematically calculate equilibria in binding reaction network models. Equilibria and other related properties are used to choose drugs with more favorable properties to advance into drug development, so an algorithmic approach would be highly desirable. The second open problem is to determine when non-monotonic responses to different drug levels can occur, by analyzing network systems. Compounds with poorly understood dose response may be discarded unnecessarily, despite other favorable properties. Anticipating possible non-monotonic dose–response curves can prevent the discarding of drugs that can be developed further. The paper of Durón et al. (2018) focuses on identifying potential drug targets using network complexity measures. In particular, Durón et al. show how analysis of gene network properties such as variability of betweenness centrality can predict functional information about genes.

Mackey and Maini (2015) asked “What has mathematics done for biology?” Here, we ask, “What can mathematics do for drug development?” There are many ways to answer this question, and we expect most ways have yet to be discovered. Our perspectives article gives multiple answers, and each of the other papers in this issue answers the question in a different way. Many of the papers in JPKPD and CPT:PSP provide additional answers. We hope to see further work in this journal and in other venues contributing more progress on this question.
